# Supplementary material for: Variation in the leaf and root microbiome of sugar maple (Acer saccharum) at an elevational range limit
Source: PeerJ. 2018 Aug 14;6:e5293. doi: 10.7717/peerj.5293 (PMC6097496; doi:10.7717/peerj.5293)
Supplement: Supplemental Information 1 — Figures S1–S2 and Tables S1–S3 [file peerj-06-5293-s001.docx]

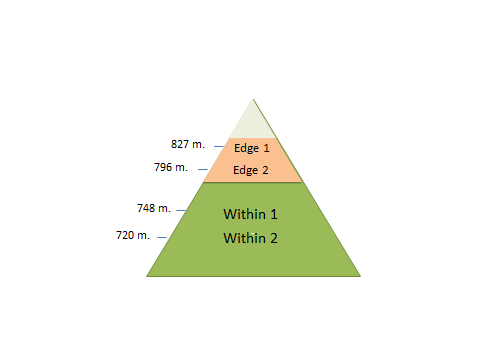


**Figure S1.** Sugar maple seedling sampling locations on Mont-Mégantic, Quebec showing the different elevations from which sugar maple seedlings were sampled along with the approximate elevation where the sugar maple elevational range edge occurs.

B

A

**Figure S2.** Operational taxonomic unit (OTU; 97% sequences similarity) collector’s curve (mean with 95% confidence intervals) based on random sampling of data before rarefaction with no singletons a) bacterial samples and b) fungal samples.

**Table S1.** Metadata relative to the plots and sugar maple seedlings taken at each plot at [Mont-Mégantic](http://www.astrolab-parc-national-mont-megantic.org/en/), Quebec.

| **Zone Name** | **Plot** | **Elevation (m.a.s.l)** | **Seedling Samples** | **From Tree Line** | **Sapling**  **Age (y)** | **SLA** | **Stem**  **Density**  **(g/cm^3^)** |
| --- | --- | --- | --- | --- | --- | --- | --- |
| Edge | 1 | 827 | 10 | Yes | 2 | 61.2 | 0.57 |
|  | 2 | 796 | 10 | Yes | 2 | 82.2 | 0.62 |
| Within | 1 | 748 | 10 | No | 3 | 83.7 | 0.52 |
|  | 2 | 720 | 10 | No | 4 | 69.1 | 0.48 |

**Table S2.** 16S rRNA and ITS samples obtained from [Mont-Mégantic](http://www.astrolab-parc-national-mont-megantic.org/en/) after sequencing which were used for analysis.

|  | **Primers** | **Within** | **Edge** | **Total** |
| --- | --- | --- | --- | --- |
| Rhizosphere | 16S | 15 | 19 | 34 |
| Root Endophytes | 16S | 10 | 19 | 29 |
| Phyllosphere | 16S | 15 | 18 | 33 |
| Leaf Endophytes | 16S | 12 | 8 | 20 |
| **Total Bacteria** | **16S** | **52** | **64** | **116** |
| Root Endophytes | ITS | 10 | 18 | 28 |

**Table S3.** Indicator taxa found at the phyla and class levels for comparisons between epiphytic bacterial communities to endophytic and between leaf and root-associated bacterial communities of sugar maple. Bacterial phyla are represented in bold text while classes are represented in italics.

|  | Epiphytic | Endophytic | Leaf | Root |
| --- | --- | --- | --- | --- |
| **PHYLA** | **Acidobacteria** |  | **Bacteroidetes** | **Acidobacteria** |
|  | **AD3** |  | **FBP** | **Actinobacteria** |
|  | **Elusimicrobia** |  | **Proteobacteria** | **Armatimonadetes** |
|  | **Firmicutes** |  | **Thermi** | **Chlamydiae** |
|  | **Gemmatimonadetes** |  |  | **Chlorobi** |
|  | **TM7** |  |  | **Elusimicrobia** |
|  | **Verrucomicrobia** |  |  | **Fibrobacteres** |
|  |  |  |  | **Gemmatimonadetes** |
|  |  |  |  | **Spirochaetes** |
|  |  |  |  | **TM6** |
|  |  |  |  | **Verrucomicrobia** |
| *CLASSES* | *ABS 6* | *Actinobacteria* | *Alphaproteobacteria* | *Acidimicrobiia* |
|  | *Acidobacteriia* | *Betaproteobacteria* | *Cytophagia* | *Acidobacteriia* |
|  | *Acidimicrobiia* |  | *Deinococci* | *Actinobacteria* |
|  | *Bacilli* |  | *Flavobacteriia* | *Armatimonadia* |
|  | *Clostridia* |  |  | *At12OctB3* |
|  | *DA052* |  |  | *Chlamydiia* |
|  | *Deltaproteobacteria* |  |  | *DA052* |
|  | *Elusimicrobia* |  |  | *Elusimicrobia* |
|  | *Ktedonobacteria* |  |  | *Fibrobacteria* |
|  | *Gemm 1* |  |  | *Fimbriimonadia* |
|  | *Gemmatimonadetes* |  |  | *Gemmatimonadetes* |
|  | *Pedosphaerae* |  |  | *Pedosphaerae* |
|  | *SC3* |  |  | *Saprospirae* |
|  | *Solibacteres* |  |  | *SC3* |
|  | *TK17* |  |  | *SJA 4* |
|  | *TM1* |  |  | *Solibacteres* |
|  | *TM7 3* |  |  | *Spirochaetes* |
|  |  |  |  | *Thermoleophilia* |
|  |  |  |  | *TM1* |
|  |  |  |  | *TM7 1* |
